# New norm values of the brief resilience scale (BRS) from the German general population with new post-COVID-19 data

**DOI:** 10.1186/s40359-024-01995-0

**Published:** 2024-09-27

**Authors:** Christoph Rösner, Elmar Brähler, Cedric Sachser, Vera Clemens, Katja Petrowski

**Affiliations:** 1 Medical Psychology and Medical Sociology, University Medical Center of the Johannes Gutenberg University, Mainz, Germany; 2 Department of Psychosomatic Medicine and Psychotherapy, University Medical Center of the Johannes Gutenberg University Mainz, Untere Zahlbacher Str. 8, 55131 Mainz, Germany; 3 Department of Medical Psychology and Medical Sociology, University of Leipzig Medical Center, Philipp-Rosenthal-Str. 55, 04103 Leipzig, Germany; 4 Department of Child and Adolescent Psychiatry/Psychotherapy, Ulm University, Steinhövelstrasse 5, 89073 Ulm, Germany; 5 Department of Internal Medicine III, University Medical Center Carl Gustav Carus, University of Dresden, Dresden, Germany

**Keywords:** BRS, German version, Representative sample, Norm data, Covid-19 pandemic

## Abstract

**Background:**

The concept of psychological resilience has spurred extensive research across various fields, with the Brief Resilience Scale (BRS) emerging as a concise tool to measure an individual’s ability to rebound from stress. It has been translated into multiple languages, including German, but the validation of the German version occurred prior to the COVID-19 pandemic. Therefore, the main objective of this study was to examine new norm values and psychometric properties of the BRS.

**Methods:**

Norm values, the factor structure, reliability and validity of the BRS were examined using data from a representative survey of the German general population (*N* = 2522).

**Results:**

The results indicated that the method-factor model showed the best fit, suggesting a nuanced understanding of resilience beyond a single-factor approach. The BRS demonstrated good convergent and discriminant validity based on both latent and manifest correlations. Moreover, the study revealed increasing postpandemic resilience scores.

**Conclusions:**

The results of this study provide support for the psychometric reliability and validity of the German version of the BRS after the COVID-19 pandemic and underscore the importance of assessing resilience amidst evolving societal challenges and highlight the need for further exploration in diverse populations.

**Supplementary Information:**

The online version contains supplementary material available at 10.1186/s40359-024-01995-0.

## Introduction

In recent decades, the idea of psychological resilience has sparked significant research across various fields, like neurobiology [1–4] and sex sciences [5, 6]. The term “resilience” denotes the phenomenon where individuals manage to sustain their mental well-being or experience only temporary mental distress despite enduring significant adversity [2, 7–9]. In terms of assessing resilience, various approaches have been developed, including more intricate concepts such as the stressor reactivity score (SR score) [10] and the resilience shield model [11], along with several questionnaires. These instruments predominantly adopt either a trait-oriented perspective, like the dispositional resilience scale (DRS) [12], or focus on gauging the availability of resources and protective factors for maintaining or regaining mental well-being despite adversities, such as the Connor-Davidson Resilience Scale (CD-RISC) [13]. Specifically targeting resilience itself— an individual’s capacity to rebound from stress despite significant adversity— Smith and colleagues introduced the Brief Resilience Scale (BRS) [14]. Within the resilience process, the BRS seem to be the only scale measuring the belief of an individual to bounce back from stress, whereas other scales, such as the CD-RISC or Resilience Scale for adults (RSA) [15], cover other aspects taking part in the resilience process, including social competence, self-efficacy and having secure relationships. This theoretical derivation of different resilience concepts for the aforementioned questionnaires has already been demonstrated empirically for the CD-RISC and BRS [16]. For an overview of other resilience questionnaires, see Salisu and Hashim [17]. Nowadays, resilience is understood as a dynamic concept, captured as an outcome of the relationship between the number and perceived exposure to stressors and the resulting reaction or symptomatics (SR score [10]), with the items of the BRS aiming to predict this outcome (e.g., ‘I tend to bounce back quickly after hard times’) [14].

The BRS [14] is a concise six-item tool designed to evaluate an individual’s capacity to rebound from stress, utilizing a five-point Likert scale (Table 1). It has favorable psychometric properties, including strong internal consistency and retest reliability. The unidimensional nature of the BRS makes it ideal for specifically measuring resilience with minimal complexity, thereby avoiding the confounding effects of multiple variables. However, this also limits its scope compared to other resilience questionnaires [17]. The BRS exhibits associations with various resilience measures (e.g., CD-RISC [13, 16]), personal attributes (e.g., optimism as assessed by the Life Orientation Test-Revisited [LOT-R] [18]), health-related outcomes (e.g., depression measured by the Hospital Anxiety and Depression Scale [HADS] [19] and postnatal depression measured by the Edinburgh Postnatal Depression Scale [EPDS] [20]), workability [21], coping strategies (e.g., active coping evaluated by the Brief COPE [22]), and social dynamics (e.g., social support gauged by the Interpersonal Support Evaluation List [ISEL] [23]). In addition, the BRS is also used to test the success of interventions to increase resilience [24, 25], for example it can be used in intervention studies both pre- and post-intervention to measure changes in the belief in one’s ability to cope with future stressors, and it is used to further understand resilience in specific jobs that frequently experience adversities, such as soldiers and healthcare workers [26, 27]. Among other factors, the BRS was used to differentiate between patients with fibromyalgia and healthy individuals [14], and the BRS moderated the occurrence of pain and general psychopathology with regard to traumatic experiences in childhood [28, 29]. To date, the BRS has been translated into a number of languages, such as Arabic [30], Korean [31], Dutch [32], Vietnamese [33], Spanish [34], and Malaysian [35]. The validation studies showed adequate psychometric qualities, with Cronbach’s α ranging from 0.81 ± 98 except for the Vietnamese version, which had a Cronbach’s α of 0.66, and convergent (i.e., correlation of BRS score ≥ 0.30 with conceptually similar measure) as well as discriminant predictive validity (i.e., correlation of BRS score ≤ 0.30 with theoretically distinct measure). Although resilience has been measured separately across different countries, few studies to date have examined the influence of cultural factors on resilience [36]. This examination is necessary to consider all facets of resilience [37]. The BRS was also translated into German, but the data collection took place from 2015 to 2016, before the coronavirus pandemic [38, 39]. The reliability of the German version was good, with α = 0.85 and ω = 0.85. For the German sample, significant correlations were calculated as follows: *r* = − .53 with perceived stress, *r* = .51 with optimism, *r* = .51 with self-efficacy, *r* = .45 with internal locus of control, and *r* = − .45 with external locus of control. The calculation of the factor structure of the German translation was carried out on a sample size of *N* = 2609^39^; however, the calculation of the norm values and of the convergent and discriminant validity was only carried out on part of this sample of *N* = 1128^40^.

**Table 1:** BRS item content and descriptive statistics

BRS Item	Item (German/Englisch)	M	SD	Cronbach’s α if item dropped	Skewness	Kurtosis
1	Ich neige dazu, mich nach schwierigen Zeiten schnell zu erholen/I tend to bounce back quickly after hard times	3.62	0.98	0.838	-0.448	-0.264
2	Es fällt mir schwer, stressige Situationen durchzustehen/I have a hard time making it through stressful events (Revised)	3.51	1.09	0.854	-0.385	-0.599
3	Ich brauche nicht viel Zeit, um mich von einem stressigen Ereignis zu erholen/It does not take me long to recover from a stressful event	3.40	1.08	0.862	-0.331	-0.623
4	Es fällt mir schwer zur Normalität zurückzukehren, wenn etwas Schlimmes passiert ist/It is hard for me to snap back when something bad happens (Revised)	3.49	1.09	0.847	-0.400	-0.577
5	Normalerweise überstehe ich schwierige Zeiten ohne größere Probleme/I usually come through difficult times with little trouble	3.63	0.97	0.839	-0.506	-0.185
6	Ich brauche tendenziell lange, um über Rückschläge in meinem Leben hinwegzukommen/I tend to take a long time to get over set-backs in my life (Revised)	3.59	1.08	0.838	-0.445	-0.519

The COVID-19 outbreak precipitated global macrostresses, such as the imposition of physical distancing measures through lockdowns, prompting significant research into resilience. The COVID-19 pandemic, like the H1N1 pandemic [40], has caused uncertainty and future-related concerns, leading to symptoms such as intrusions, avoidance behavior, and hyperarousal, thus prompting debate on its classification as a traumatic stressor [41]. As a result of dealing with the stressor of the COVID-19 pandemic, individuals’ beliefs about their resilience might have changed; for instance, someone who successfully coped with the pandemic stressor might now have a higher belief in their ability to handle future stressors. In Germany, lockdowns were instituted by the government on 22.03.2020 and on 02.11.2020. With depression and anxiety levels, measured by Patient Health Questionnaire for Depression and Anxiety (PHQ-4), rising in the German population in 2020 (M = 2.45) and 2021 (M = 2.21) (peripandemic) compared to those in 2019 (M = 1.79) (prepandemic) [42], researchers have investigated both risk factors and protective factors. Among these, resilience, assessed using the BRS, among other metrics, did not appear to influence the biological stress response during the lockdown period [43], as measured by hair cortisol levels. Protective factors often include resilience-related traits such as optimism and self-efficacy alongside the resilience mechanism of a positive evaluation style postulated by Kalisch et al. [2, 27, 42, 40, 41]. Although psychological resilience to adversity generally did not seem to affect coping with the macrostressor of the COVID-19 pandemic, findings from the initial COVID-19 pandemic wave in spring 2020 indicated higher resilience scores among healthcare workers than among the general population before the pandemic [27]. Altered BRS values, potentially even higher scores, may have resulted from the COVID-19 pandemic in Germany, as the pandemic’s impact varied across the population and did not inherently produce negative effects [44, 45].

The objective of the present study was to assess the reliability and validity of the German translation of the BRS as well as its norm values with the most recent representative data set. Based on the findings from the aforementioned translated versions of the BRS, three models appear to be relevant for the factor structure of the BRS: a one-factor model, a two-factor model, and a method factor model [30–35, 38]. It is hypothesized that the best model fit results for the method factor model. In line with the findings of Smith and colleagues [14] and Kunzler et al. [39]. , our hypothesis suggests negative correlations between BRS and anxiety, severe depression and external locus of control and positive correlations between BRS and resilience factors, such as internal locus of control and self-efficacy. Furthermore, based on the results of Kunzler et al. [39], we hypothesize that males will achieve higher BRS values compared to females, and that BRS values will decrease with increasing age.

## Method

### Participants and procedures

The present study was approved by the ethics committee of the University of Leipzig (594/21-ek). The participants were informed about the anonymization of all personal data, the study procedures, and the data collection. The participants provided verbal informed consent according to German law, which was documented by a research associate before starting the survey. The present study is based on a representative survey of the German general population. The data were collected in 2022 by the Independent Service for Surveys, Methods and Analyses (USUMA, Berlin) using the random-route technique. Comparing the present sample with information from the Federal Statistical Office, it may be assumed that the sample is fairly representative of the general population concerning age and gender, with a lower proportion of participants aged 29 or younger and a slightly greater proportion of all remaining age groups represented in the study sample (Federal Statistical Office, 2023). This demographic distribution might result in an overall lower mean value of the BRS for our sample, as higher BRS values were observed for the younger age group in 2016^47^. The questionnaire was personally handed out by a research associate at the participants’ homes, and the participants then completed it independently. The first attempt to contact the participants was made for 4,386 addresses, of which 4,360 were valid. Out of the initial sample, the study sample consisted of 2,522 men, women and nonbinary persons (participation rate: 57.8% of valid addresses). When persons with missing data were excluded, the sample included *N* = 2503 individuals (0.008% with missing data). As full information maximum likelihood method was used for confirmatory factor analysis, *N* = 2,522 is given in some places [46]. The percentage of women was 50.1% (*N* = 1,264). As only four people stated that they were nonbinary, it was not possible to calculate norm values for this group. In a representative health survey of the German population in 2019, with a sample size of 23,001 people, 0.13% of respondents identified as non-binary [47]. This proportion is quite close to 0.16% of our sample. However, it has not yet been possible to conclusively determine the proportion of non-binary individuals in the German population. Consequently, the impact of the four non-binary participants on the representativeness of the study results cannot be definitively clarified. Further characteristics of the study sample are given in Table 2.

**Table 2:** Demographic characteristics of the sample

Variable	*n*	Percentage
Gender		
Female	1264	50.1
Divers	4	0.2
Age		
< 25	209	8.3
25–34	408	16.2
35–44	451	17.9
45–54	417	16.5
55–64	489	19.4
65–74	341	13.5
> 75	207	8.2
Education		
No formal degree	60	2.4
≤ 9 years (Hauptschule) or ≤ 10 years (Realschule) without degree	620	24.6
≤ 10 years (Realschule)	1057	42
≤ 12 years (Subject-linked) university entrance qualification	331	13.1
University of applied sciences or university degree	402	16
Other	48	1.9
Employment		
Yes	1629	64
No	902	35.9
Religion		
Protestant	873	34.9
Catholic	723	28.9
Muslim	62	2.5
Other	46	1.8
without confession	801	32.0
Weekly working time (hours)		
< 15	61	2.4
15–34	304	12.1
> 34	1224	48.7
Relationship status		
married/living together	1165	46.2
married/not living together	41	1.6
single	736	29.2
divorced	363	14.4
widowed	215	8.5
Born		
in East Germany	584	23.5
in West Germany	1776	71.5
Abroad	124	5.0
ASKU (M/SD)	2512	3.96 (0.77)
PHQ2 (M/SD)	2515	1.39 (0.58)
GAD2 (M/SD	2514	1.32 (0.55)
Weight (kg)	2522	100

### Instruments

We assessed stress resilience using the German version of the Brief Resilience Scale (BRS), as described by Chmitorz, Wenzel, et al. [38]. and Kunzler et al. [39]. , which consists of six items rated on a 5-point Likert scale. Notably, negatively phrased items underwent recoding for the mean calculation (items 2, 4 and 6; see Table 1). Higher scores on this scale indicate greater stress recovery ability, with prior research validating its psychometric properties [38, 39].

The self-report inventory, called the Assessment of Self-Efficacy (ASKU), consists of a short German version of three positively framed items assessing self-efficacy on a 5-point Likert scale [48]. The ASKU had robust internal consistency (ω = 0.81–0.86; α NR), and the scale exhibited associations with life satisfaction, optimism, locus of control, and another self-efficacy scale.

The four-item Internal-External Locus of Control Scale (IE-4) evaluates beliefs about internal and external control measured on a 5-point Likert scale [49]. Kovaleva [49] reported moderate to acceptable reliability for two subscales (internal locus of control [IEint]: ω = 0.70–0.71; external locus of control [IEext]: ω = 0.53–0.63), with correlations with life satisfaction, optimism, and self-efficacy.

The PHQ-4, which starts with an introductory question, includes two anxiety and two depression items, each scored from 0 to 3 [52]. The cumulative scores range from 0 to 12, with the recommended cutoffs for the PHQ-2 and GAD-2 suggested by Kroenke et al. [50, 51]. Recent research confirmed the acceptable reliability of the German version of the PHQ-4 (ω = 0.85) and its subscales PHQ-2 (ω = 0.77) and GAD-2 (ω = 0.78) in a large sample [52].

### Statistical analysis

The primary analyses conducted in this study encompassed the examination of the factor structure, reliability, validity, and norm values associated with the Brief Resilience Scale (BRS). To assess the factor structure’s validity, we utilized confirmatory factor analyses employing maximum likelihood estimation and covariance matrices. Drawing from prior research [30–35, 38], we tested three models: (1) a one-factor model representing general resilience, (2) a two-factor model categorizing positively and negatively worded items (items 1, 3, 5; items 2, 4, 6, respectively), and (3) a two-factor model incorporating general resilience (items 1, 2, 3, 4, 5, 6) along with a method factor accounting for the positive and negative wording of items (items 2, 4, 6). In accordance with Hu and Bentler’s guidelines [53], we evaluated and compared the fit of these models using the chi-square test, root mean square error of approximation (RMSEA), comparative fit index (CFI), and standardized root mean squared residual (SRMR). Values of 0.08 or lower for RMSEA, 0.95 or higher for CFI, and 0.06 or lower for SRMR are typically considered indicative of acceptable fit. The likelihood ratio (LR) test was employed to assess the fit of the three models, where a statistically significant result implies that the fit of the alternative model surpasses that of the prior (null) model. We assessed measurement invariance across age, gender, and BMI (kg/m²) status following the procedure outlined by Milfont and Fischer [54]. This involved comparing increasingly constrained models in a stepwise manner to establish progressively stricter levels of invariance. First, we evaluated metric (or weak) invariance by comparing the unconstrained model to one where factor loadings were constrained to be equal across groups. Next, we tested scalar (or strong) invariance by comparing the metric model to a model with additional constraints on item intercepts. Finally, we assessed strict invariance by comparing the scalar model to one where residuals were also constrained to be equal across the groups. To evaluate reliability, we computed not only Cronbach’s α but also estimated reliability using McDonald’s omega (ω). Convergent and discriminant validity were established through correlation analysis, examining the relationships between the BRS and related constructs such as self-efficacy (ASKU [48]), internal locus of control (IE_inter_), external locus of control (IE_exter_) [49], which were also used in the prior validation of the German version of the BRS [39] and divergent constructs such as depression (PHQ-2) and anxiety (GAD-2) [55]. In our study, the correlation coefficients were interpreted using Cohen’s [56] guidelines for the magnitude of correlations, where values of approximately 0.10 are considered small, around 0.30 are considered moderate, and those of 0.50 or above are considered large. The construct validity of the BRS was established through confirmatory factor analysis with regard to internal and external locus of control and self-efficacy. Four different models were compared, with a unidimensional model for all items, a two-factor model with one factor for BRS, ASKU and IE_inter_ and one factor for IE_exter_, a three-factor model with one factor for BRS and ASKU, one factor for IE_inter_ and one factor for IE_exter_ and a four-factor model with four separate factors accounting for the items of BRS, ASKU, IE_inter_ and IE_exter_. The comparison of these models was based on the aforementioned fit indices for the factor structure of the BRS. ANOVAs were conducted to examine the influence of various sociodemographic variables on BRS scores. The statistical analysis was carried out using Jamovi [57], Lavaan [58], R Version 4.4.0, semTools [59] and SPSS Version 23.

## Results

Mean values and standard deviations of all BRS items are provided in Table 1. All items exhibit a slight to moderate negative skew, meaning that the responses tend to cluster towards the higher end of the scale. The kurtosis values indicate that the distributions are generally flatter than a normal distribution, suggesting a more even spread of responses across the scale, rather than a pronounced peak (Table 1).

### Factor structure

As indicated in Table 3, the two-factor model (Model 2) fit the data significantly better than did the one-factor model (Model 1) (Δχ2 = 730.24, *p* < .001). The method-factor (Model 3) model also fit the data significantly better than the one-factor model (Model 1) (Δχ2 = 725.74, *p* < .001). There is no significant difference between Model 2 and Model 3, even if the chi-squared value of Model 2 is slightly lower (Δχ2 = 4.49).

**Table 3:** Factor structure of the BRS. The results from CFAs

	*n*	χ²	df	*p*	RMSEA	CFI	SRMR	AIC
One Factor	2522	789.200	9	< 0.001	0.186	0.889	0.065	37752.649
Two Factors	2522	58.963	8	< 0.001	0.050	0.993	0.020	37024.412
Two Factors (Method)	2522	63.456	8	< 0.001	0.053	0.992	0.019	37028.905

Theoretically derived, the good fit of the Model 2 model might also result from a wording effect of positively and negatively formulated items, as explained above. As the mentioned effect is taken into account in the method-factor model, this model is assessed here as the better of the two. In their statistical comparisons of different questionnaires, Schmalbach et al. [60]. also came to the conclusion that it is advisable to prefer the method-factor model in the case of wording effects. The overall model fit of the method-factor model (model 3) was excellent, as indicated by other model fit indices (RMSEA = 0.053, CFI = 0.992, SRMR = 0.019). The two-factor model (Model 2) achieved values similar to those of Model 3 for the other indices (Table 3). Given the excellent fit of the method-factor model (Model 3), we opted to analyze the reliability, validity and group differences for this model.

To evaluate measurement invariance of the BRS scores, we conducted a series of increasingly stringent tests: configural, metric, and scalar invariance. First, the measurement invariance to gender is reported, then to age. The configural invariance model, which allows all parameters to vary across groups, provided a good fit to the data: χ²(16) = 74.910, *p* < .001. The fit indices were strong, with a Comparative Fit Index (CFI) of 0.991 and a Tucker-Lewis Index (TLI) of 0.984. The Root Mean Square Error of Approximation (RMSEA) was 0.054, with a 90% confidence interval of 0.042 to 0.067, and the Standardized Root Mean Square Residual (SRMR) was 0.018. Next, we tested metric invariance by constraining the factor loadings to be equal across gender groups. The model fit remained strong: χ²(22) = 83.236, *p* < .001, with fit indices showing a slight improvement in TLI (0.988) while maintaining the CFI at 0.991. The RMSEA was 0.047 (90% CI: 0.037 to 0.058), and the SRMR increased slightly to 0.034. Finally, we tested scalar invariance by additionally constraining item intercepts to be equal across gender groups. The scalar invariance model also demonstrated a good fit: χ²(26) = 87.212, *p* < .001, with CFI and TLI values of 0.991 and 0.990, respectively. The RMSEA was further reduced to 0.043 (90% CI: 0.034 to 0.054), and the SRMR remained at 0.034. The configural invariance model, which allowed all parameters to vary across age groups, showed a good fit to the data: χ²(56) = 124.279, *p* < .001. The fit indices were strong, with a Comparative Fit Index (CFI) of 0.990 and a Tucker-Lewis Index (TLI) of 0.982. The Root Mean Square Error of Approximation (RMSEA) was 0.058, with a 90% confidence interval of 0.045 to 0.072, and the Standardized Root Mean Square Residual (SRMR) was 0.021. Next, we tested metric invariance by constraining the factor loadings to be equal across age groups. The model fit remained strong: χ²(92) = 178.080, *p* < .001, with a CFI of 0.988 and a TLI of 0.986. The RMSEA was 0.051 (90% CI: 0.040 to 0.062), and the SRMR increased to 0.070. Finally, we tested scalar invariance by additionally constraining item intercepts to be equal across age groups. The scalar invariance model also demonstrated a good fit: χ²(116) = 194.102, *p* < .001, with CFI and TLI values of 0.989 and 0.990, respectively. The RMSEA was 0.043 (90% CI: 0.032 to 0.054), and the SRMR was 0.071. The measurement invariance testing across different gender and age groups revealed that the BRS scale maintains configural, metric, and scalar invariance. This indicates that the BRS scale functions equivalently across the various gender and age groups, allowing for meaningful comparisons of BRS scores across these groups.

### Reliability

Cronbach’s α showed good reliability, with α = 0.87. For McDonald’s Omega, the reliability was ω = 0.87. Table 1 lists Cronbach’s alpha values when items are dropped.

### Construct validity

According to the results presented in Table 4, the four-factor model demonstrated the best fit, whereas the one-factor model, encompassing a single general factor accounting for all items, exhibited the poorest fit to the data. Notably, compared to both the two-factor and three-factor models, the four-factor model consistently exhibited superior fit indices. For additional information on the factor loadings and the average variance extracted (AVE) of the models, please refer to Tables S1-S4.

**Table 4:** Results from CFA regarding construct validity

	*n*	χ²	df	*p*	RMSEA	CFI	SRMR	AIC
One Factor	2522	3665	65	< 0.001	0.15	0.81	0.08	75,695
Two Factors	2522	3237	64	< 0.001	0.14	0.83	0.14	75,269
Three Factors	2522	2808	62	< 0.001	0.13	0.85	0.07	74,844
Four Factors	2522	1300	59	< 0.001	0.09	0.93	0.05	73,343

One Factor: BRS, IEinter, IEexter, ASKU; Two Factors: Factor 1 = BRS, IEinter, ASKU and Factor 2 = IEexter; Three Factors: Factor 1: BRS, ASKU and Factor 2 = IEexter and Factor 3 = IEinter; Four Factors = one factor for each questionnaire

### Convergent and discriminant validity

We conducted an examination of convergent and discriminant validity by analyzing latent correlations among the Brief Resilience Scale (BRS) and various measures encompassing self-efficacy, beliefs about locus of control, depression, and anxiety. Our findings revealed significant moderate negative correlations between the BRS and symptoms of depression, symptoms of anxiety, and external locus of control. Conversely, we observed a large positive correlation between the BRS and self-efficacy as well as a moderate correlation between BRS and internal locus of control. Self-efficacy also moderately correlates with internal locus of control (IE1) and negatively with mental health symptoms. Depression and anxiety are strongly correlated with each other. External locus of control (IE2) shows weaker correlations, being moderately associated with higher depression and anxiety, and negatively with resilience and self-efficacy. All correlations demonstrated statistical significance (see Table 5). The observed correlations of the BRS with other questionnaires aligned with the anticipated directions, underscoring adequate convergent and discriminant validity of the BRS.

**Table 5:** Correlation matrix

	M	SD	BRS	IE1	IE2	ASKU	PHQ2	GAD2
BRS	3.54	0.81	**0.87**	0.588^**^	− 0.486^**^	0.721^**^	− 0.522^**^	− 0.481^**^
IE1	4.12	0.77	0.588^**^	**0.79**	− 0.394^**^	0.690^**^	− 0.448^**^	− 0.401^**^
IE2	2.34	0.87	− 0.486^**^	− 0.394^**^	**0.66**	− 0.364^**^	0.397^**^	0.475^**^
ASKU	3.96	0.77	0.721^**^	0.690^**^	− 0.364^**^	**0.90**	− 0.454^**^	− 0.453^**^
PHQ2	1.39	0.58	− 0.522^**^	− 0.448^**^	0.397^**^	− 0.454^**^	**0.83**	0.763^**^
GAD2	1.32	0.55	− 0.401^**^	− 0.401^**^	0.475^**^	− 0.394^**^	0.763^**^	.**79**

resilience (BRS), internal locus of control (IE1), external locus of control (IE2), self-efficacy (ASKU) and the two subscales of the Patient Health Questionnaire (PHQ) measuring two depressive items (PHQ2) and two anxiety items (GAD2) with Cronbach’s α values on the diagonal, **. *P* < .01

### Influence of sociodemographic variables on BRS scores

The results indicated significant differences in BRS scores across gender (F(2, 2500) = 39.4, *p* < .001, η2 = 0.031) and age groups (F(6, 2496) = 4.39, *p* < .001, η2 = 0.010). For gender, we found that women reported lower BRSs than men did (MDiff = 0.28, *p* < .001, Cohen’s d = 0.349). Since only four participants identified as nonbinary, the sample size was too small to conduct statistical tests, and their data were excluded from the analysis of gender differences. Regarding age-related differences, we found no differences between those under 25 years of age and those in other age groups. We found small effect sizes in which a lower BRS was associated with increasing age, as participants aged 25 to 34 years and 35 to 44 years reported higher BRSs than participants aged 65 to 74 years (MDiff_25 − 34_ = 0.22, *p* = .004, Cohen’s d = 0.273, MDiff_35 − 44_ = 0.20, *p* = .014, Cohen’s d = 0.242) and older than 75 years (MDiff_25 − 34_ = 0.26, *p* = .003, Cohen’s d = 0.321; M_35 − 44_ = 0.24, *p* = .010, Cohen’s d = 0.290). We also conducted a regression analysis for the influence of weight on BRS scores. Weight significantly predicted BRS values (β = -0.28, *p* < .001). Regarding the weekly working time, it was found that persons working more than 35 h had significantly greater BRS values than persons working 15 to 34 h (MDiff = 0.32, *p* < .001, Cohen’s d = 0.403) and persons working less than 15 h (MDiff = 0.55, *p* < .001, Cohen’s d = 0.710). For persons with 15 to 34 h and persons with less than 15 h, no difference was found.

### Norm values

The Tables S5-S8 contain population norms containing percent ranks and stanine values for the total sample as well as normative values stratified by age and gender.

## Discussion

The aim of the study was to evaluate new norm values of the BRS and to test its reliability and validity. As with Chmitorz et al. [38]. , the scale showed good reliability. With regard to the factor structure, the fit indices for the method factor model and the two-factor model were similarly good, but we opted for the method factor model, as explained in the results section [60]. This result was also obtained in other studies, making it advisable to calculate the method factor for the BRS in future studies to take wording effects into account [16, 34, 38]. The separation of the true variance in resilience scores from the variance attributable to item wording effects is crucial for understanding resilience because it ensures that the observed scores accurately reflect an individual’s ability to bounce back from adversity, rather than being influenced by how the questions are framed. By considering the method factor, researchers can better isolate the core construct of resilience, leading to more precise assessments and deeper insights into how resilience operates across different populations and contexts. The measurement invariance analysis demonstrated that the BRS scale achieved configural, metric, and scalar invariance across both gender and age groups, indicating that the scale functions equivalently across these groups, allowing for meaningful comparisons of resilience scores. The unidimensional nature of the BRS provides simplicity and ease of use, focusing on resilience as the ability to bounce back from adversity. This makes it ideal for quick assessments. However, it may overlook broader aspects of resilience captured by multidimensional scales. While multidimensional scales offer a more comprehensive view, they are also more complex. The choice between the BRS and a multidimensional scale should depend on the specific goals of the assessment.

Compared to that in the precoronavirus pandemic period, the mean value for the BRS increased overall (M = 3.35, SD = 0.95, M = 3.54, SD = 0.81)^39^. This could be the result of a truly greater ability to “bounce back” from stress after the coronavirus pandemic. The pandemic might have acted as a significant stressor that, paradoxically, enhanced resilience in the population. This aligns with resilience theories suggesting that exposure to adversity can strengthen resilience over time, as individuals develop coping mechanisms and adaptive strategies to deal with stress, when the stressor exposure does not exceed the individuals capacies [10, 61]. The increase of the mean BRS value could also be related to the reduction in microstressors due to the lockdown and, consequently, to recovery [45] or to increased activation of behaviors associated with resilience factors and mechanisms, such as self-efficacy, optimism and a positive evaluation style, even after the COVID-19 pandemic [27, 42]. For instance, Kalisch et al.‘s [2] theory of resilience mechanisms postulates that a positive evaluation style, which includes a tendency to see stressful events in a more favorable light, can mitigate the impact of stressors and contribute to higher resilience scores. The correlation with self-efficacy was the highest, which may have implications for the discriminant validity of the BRS. Since self-efficacy is a component of resilience, these constructs are inherently related and may be more closely linked when measured by the BRS. However, the BRS encompasses a broader construct that also includes other factors of resilience. However, this higher BRS value may also be related to a wording effect, as inverted items 2, 4 and 6 in particular increased compared to those before the pandemic [38]. Consequently, if BRS data were collected before and after a macrostressor such as the COVID-19 pandemic or similar public health crisis, it could provide valuable information about whether and how the belief in the ability to bounce back from stress changes in relation to this macrostressor.

In terms of construct validity, the four-factor model, in which resilience, self-efficacy, and internal and external locus of control each represent a separate factor, provided the best model fit. According to this result, the BRS, as the ability to recover from stress, represented an independent construct in relation to the aforementioned resilience factors. However, considering the results from the correlation analyses on convergent reliability, the degree of separation between BRSs and ASKUs seemed less clear due to the greater correlation (*r* = .72, *p* < .001). In the validation of the BRS by Kunzler et al. [39]. This correlation was smaller (*r* = .51). Compared to the results of Kunzler et al. [39]. , the mean value of ASKU in 2017 (M = 3.97) did not seem to differ from the mean value of our current study (M = 3.96). As self-efficacy is part of the multidimensional construct of resilience as a resilience factor [24], the increase in this correlation might be due to the greater importance of this multidimensional construct after the COVID-19 pandemic. In Germany, the Robert Koch Institute published the daily infection rate of COVID-19 during the coronavirus pandemic, making the efficacy of political regulations, such as wearing a mask, visible. The finding that one’s behavior may affect not only one’s own health but also society’s health might also explain the greater correlation between self-efficacy and resilience. Consequently, resilience, the ability to recover from stress, can be measured specifically by the BRS, and the influence of self-efficacy as a resilience factor on the multidimensional construct of resilience may have increased in recent years. Regarding discriminant validity, the direction of the correlation between resilience and depression and anxiety was negative, as hypothesized, and was similar to the results of the correlation of the BRS with depression and anxiety measured by Chmitorz et al. [38]. (r_depression_ = − 0.41, r_anxiety_ = − 0.45) and in the original version [14]. (r_depression_ = − 0.41 to − 0.66, r_anxiety_ = − 0.46 to − 0.60). Understanding the relationship between the Brief Resilience Scale (BRS) and mental health indicators such as depression and anxiety has significant practical implications, as it could inform the development of targeted interventions that enhance resilience while simultaneously addressing symptoms of these disorders, ultimately leading to more effective and personalized mental health care strategies.

In terms of the impact of sociodemographic variables on BRS scores, women reported lower BRS scores than men. However, it should not be prematurely concluded that male gender is a resilience factor, as co-occurring gender disparities, such as psychosocial responsibilities (e.g., caretaking), may play a more significant role [62]. A recent review by Kalisch et al. concludes that the current body of data is insufficient to definitively assess the influence of gender on resilience [63]. Age-related analysis indicated a slight decline in resilience with increasing age, particularly for those aged 65 and older. Our results align with those of Kunzler et al. [39] and contradict the socioemotional selectivity theory, which suggests that older people develop greater stress-coping capacities over time, leading to increased resilience [64]. Additionally, higher body weight was linked to lower BRS scores. Although results from the German population indicate a positive correlation between increasing BMI and anxiety and depression [65]—implying a likely negative correlation between resilience and BMI—studies in Portuguese and British samples have also found a negative correlation between resilience and BMI [66], while a Chinese sample showed a positive correlation [67]. These divergent findings underscore the need for further research on the relationship between resilience and BMI across different populations. Our findings suggest that individuals who work longer hours per week tend to have higher BRS scores, indicating greater resilience. This relationship aligns with the results of Kunzler et al. [39], who also found that increased working hours are associated with higher resilience, possibly due to the psychological benefits of sustained work engagement and a sense of accomplishment.

The strengths of this study are the use of new norm values for the German population after the coronavirus pandemic and the use of a larger and more heterogeneous sample. Furthermore, this sample was also larger for the calculation of convergent and discriminant validity than for the validation by Kunzler et al. [39]. The new norm values for the BRS have important practical implications for both clinical and research settings. In clinical practice, these updated norms provide a more accurate benchmark for assessing resilience in diverse populations, allowing for more informed and relevant interpretations. For researchers, the new norms facilitate better comparisons across studies, enhancing the consistency and applicability of resilience research. These updates ensure that the BRS remains a valuable tool for measuring resilience in contemporary contexts. The BRS has broad applicability across various settings, including clinical, organizational, and educational contexts; however, its use may be limited by factors such as reliance on self-reporting and the need for contextual adaptations to ensure validity and effectiveness in diverse populations. The limitations are that only four people with nonbinary gender identity participated; therefore, no norm values could be calculated for this group. New results on BRS scores of alternative sexuality communities in America show that they have higher scores than people with male and female gender identity [6]. Future German studies with the BRS should also look at these groups. In addition, only the BRS alone was used as a resilience questionnaire, so no comparison with other resilience questionnaires is possible, as suggested by Yun et al. [16]. Future research should include test-retest reliability assessments to provide a more comprehensive understanding of the temporal stability of resilience. Such studies would help determine how resilience changes over different time periods and under varying circumstances, thereby enhancing the validity and applicability of resilience scales. Another limitation of this study is the exclusive reliance on self-report measures, specifically the Brief Resilience Scale (BRS), for assessing resilience. This reliance on self-report measures limits our ability to capture more nuanced or unconscious aspects of resilience that might be better assessed through other methods, such as behavioral observations or physiological measurements. Future research should aim to incorporate a broader range of assessment tools to complement self-report measures, which would offer a more holistic understanding of resilience and potentially address the limitations associated with self-report data. While our study provides valuable insights into the normative values and factorial structure of the BRS, it does not include analyses of potential moderating or mediating effects, such as those of age or gender, nor does it incorporate Item Response Theory (IRT) analysis. These advanced analyses, though beyond the scope of the current paper, represent important avenues for future research that could further deepen our understanding of resilience and the functioning of the BRS across different populations. Furthermore, future research could benefit from analyzing extreme groups, such as individuals with high and low resilience, to gain deeper insights into the factors specifically associated with these subgroups and to enhance our understanding of resilience dynamics across different population segments. Future research should investigate cross-cultural influences on resilience to comprehensively capture all dimensions of this construct [37].

In conclusion, our study validated the use of the Brief Resilience Scale (BRS) within the German population, particularly during the COVID-19 pandemic. We found robust psychometric properties, with the method-factor model emerging as the most suitable structure. Postpandemic, resilience scores increased, suggesting potential shifts in coping mechanisms or wording effects. Further research should explore resilience dynamics in diverse populations to deepen our understanding of this complex phenomenon.

## Electronic supplementary material

Below is the link to the electronic supplementary material.

Supplementary Material 1

Supplementary Material 2

## Data Availability

The datasets analysed during the current study are not publicly available but are available from the corresponding author on reasonable request.
