# Supplementary material for: New norm values of the brief resilience scale (BRS) from the German general population with new post-COVID-19 data
Source: BMC Psychol. 2024 Sep 27;12:499. doi: 10.1186/s40359-024-01995-0 (PMC11437619; doi:10.1186/s40359-024-01995-0)
Supplement: Supplementary file 1 — Supplementary Material 1 [file 40359_2024_1995_MOESM1_ESM.docx]

**Construct validity: factor loadings of the CFAs**

Table S1. One-Factor model (BRS, IEinter, IEexter, ASKU).

| **Factor** | | **Indicator** | | **Estimate** | | **SE** | | **Z** | | **p** | | **Stand. Estimate** | | **AVE** |
| --- | --- | --- | --- | --- | --- | --- | --- | --- | --- | --- | --- | --- | --- | --- |
| Factor 1 |  | BRS1 |  | 0.806 |  | 0.0173 |  | 46.6 |  | < .001 |  | 0.806 |  | 47% |
|  |  | nBRS2 |  | 0.664 |  | 0.0210 |  | 31.6 |  | < .001 |  | 0.592 |  |  |
|  |  | BRS3 |  | 0.668 |  | 0.0208 |  | 32.1 |  | < .001 |  | 0.614 |  |  |
|  |  | nBRS4 |  | 0.678 |  | 0.0210 |  | 32.3 |  | < .001 |  | 0.604 |  |  |
|  |  | BRS5 |  | 0.763 |  | 0.0176 |  | 43.3 |  | < .001 |  | 0.769 |  |  |
|  |  | nBRS6 |  | 0.739 |  | 0.0202 |  | 36.6 |  | < .001 |  | 0.666 |  |  |
|  |  | IE1 |  | 0.550 |  | 0.0157 |  | 35.1 |  | < .001 |  | 0.659 |  |  |
|  |  | IE2 |  | 0.630 |  | 0.0161 |  | 39.1 |  | < .001 |  | 0.714 |  |  |
|  |  | IE3 |  | -0.414 |  | 0.0211 |  | -19.6 |  | < .001 |  | -0.402 |  |  |
|  |  | IE4 |  | -0.449 |  | 0.0204 |  | -22.0 |  | < .001 |  | -0.445 |  |  |
|  |  | ASKU1 |  | 0.695 |  | 0.0148 |  | 46.9 |  | < .001 |  | 0.810 |  |  |
|  |  | ASKU2 |  | 0.716 |  | 0.0145 |  | 49.5 |  | < .001 |  | 0.839 |  |  |
|  |  | ASKU3 |  | 0.734 |  | 0.0153 |  | 48.1 |  | < .001 |  | 0.824 |  |  |

Table S2. Two-Factor model (Factor 1 = BRS, IEinter, ASKU and Factor 2 = IEexter).

| **Factor** | | **Indicator** | | **Estimate** | | **SE** | | **Z** | | **p** | | **Stand. Estimate** | | **AVE** |
| --- | --- | --- | --- | --- | --- | --- | --- | --- | --- | --- | --- | --- | --- | --- |
| Factor 1 |  | BRS1 |  | 0.807 |  | 0.0173 |  | 46.6 |  | < .001 |  | 0.807 |  | 52% |
|  |  | nBRS2 |  | 0.660 |  | 0.0210 |  | 31.4 |  | < .001 |  | 0.588 |  |  |
|  |  | BRS3 |  | 0.669 |  | 0.0208 |  | 32.1 |  | < .001 |  | 0.615 |  |  |
|  |  | nBRS4 |  | 0.674 |  | 0.0210 |  | 32.1 |  | < .001 |  | 0.600 |  |  |
|  |  | BRS5 |  | 0.763 |  | 0.0176 |  | 43.3 |  | < .001 |  | 0.769 |  |  |
|  |  | nBRS6 |  | 0.735 |  | 0.0202 |  | 36.3 |  | < .001 |  | 0.662 |  |  |
|  |  | IE1 |  | 0.549 |  | 0.0157 |  | 35.0 |  | < .001 |  | 0.657 |  |  |
|  |  | IE2 |  | 0.630 |  | 0.0161 |  | 39.1 |  | < .001 |  | 0.714 |  |  |
|  |  | ASKU1 |  | 0.697 |  | 0.0148 |  | 47.1 |  | < .001 |  | 0.812 |  |  |
|  |  | ASKU2 |  | 0.718 |  | 0.0144 |  | 49.8 |  | < .001 |  | 0.842 |  |  |
|  |  | ASKU3 |  | 0.737 |  | 0.0152 |  | 48.4 |  | < .001 |  | 0.827 |  |  |
| Factor 2 |  | IE3 |  | 0.684 |  | 0.0248 |  | 27.6 |  | < .001 |  | 0.665 |  | 50% |
|  |  | IE4 |  | 0.748 |  | 0.0251 |  | 29.8 |  | < .001 |  | 0.742 |  |  |

Table S3. Three-Factor model (Factor 1: BRS, ASKU and Factor 2 = IEexter and Factor 3 = IEinter).

| **Factor** | | **Indicator** | | **Estimate** | | **SE** | | **Z** | | **p** | | **Stand. Estimate** | | **AVE** |
| --- | --- | --- | --- | --- | --- | --- | --- | --- | --- | --- | --- | --- | --- | --- |
| Factor 1 |  | BRS1 |  | 0.816 |  | 0.0173 |  | 47.2 |  | < .001 |  | 0.815 |  | 58% |
|  |  | nBRS2 |  | 0.666 |  | 0.0210 |  | 31.7 |  | < .001 |  | 0.594 |  |  |
|  |  | BRS3 |  | 0.678 |  | 0.0208 |  | 32.5 |  | < .001 |  | 0.622 |  |  |
|  |  | nBRS4 |  | 0.681 |  | 0.0210 |  | 32.4 |  | < .001 |  | 0.607 |  |  |
|  |  | BRS5 |  | 0.771 |  | 0.0176 |  | 43.9 |  | < .001 |  | 0.776 |  |  |
|  |  | nBRS6 |  | 0.742 |  | 0.0202 |  | 36.7 |  | < .001 |  | 0.668 |  |  |
|  |  | ASKU1 |  | 0.693 |  | 0.0149 |  | 46.5 |  | < .001 |  | 0.807 |  |  |
|  |  | ASKU2 |  | 0.716 |  | 0.0145 |  | 49.3 |  | < .001 |  | 0.839 |  |  |
|  |  | ASKU3 |  | 0.736 |  | 0.0153 |  | 48.2 |  | < .001 |  | 0.826 |  |  |
| Factor 2 |  | IE3 |  | 0.695 |  | 0.0250 |  | 27.8 |  | < .001 |  | 0.675 |  | 50% |
|  |  | IE4 |  | 0.736 |  | 0.0251 |  | 29.3 |  | < .001 |  | 0.731 |  |  |
| Factor 3 |  | IE1 |  | 0.655 |  | 0.0156 |  | 42.1 |  | < .001 |  | 0.785 |  | 67% |
|  |  | IE2 |  | 0.751 |  | 0.0161 |  | 46.6 |  | < .001 |  | 0.852 |  |  |

Table S4. Four-Factor model (Four Factors = one factor for each questionnaire).

| **Factor** | | **Indicator** | | **Estimate** | | **SE** | | **Z** | | **p** | | **Stand. Estimate** | | **AVE** |
| --- | --- | --- | --- | --- | --- | --- | --- | --- | --- | --- | --- | --- | --- | --- |
| Factor 1 |  | BRS1 |  | 0.834 |  | 0.0174 |  | 48.0 |  | < .001 |  | 0.832 | 54% | |
|  |  | nBRS2 |  | 0.741 |  | 0.0207 |  | 35.7 |  | < .001 |  | 0.661 |  | |
|  |  | BRS3 |  | 0.724 |  | 0.0207 |  | 34.9 |  | < .001 |  | 0.664 |  | |
|  |  | nBRS4 |  | 0.777 |  | 0.0206 |  | 37.7 |  | < .001 |  | 0.692 |  | |
|  |  | BRS5 |  | 0.808 |  | 0.0174 |  | 46.4 |  | < .001 |  | 0.812 |  | |
|  |  | nBRS6 |  | 0.826 |  | 0.0198 |  | 41.8 |  | < .001 |  | 0.744 |  | |
| Factor 2 |  | IE3 |  | 0.689 |  | 0.0243 |  | 28.4 |  | < .001 |  | 0.669 | 49% | |
|  |  | IE4 |  | 0.744 |  | 0.0244 |  | 30.5 |  | < .001 |  | 0.738 |  | |
| Factor 3 |  | IE1 |  | 0.653 |  | 0.0153 |  | 42.6 |  | < .001 |  | 0.786 | 67% | |
|  |  | IE2 |  | 0.744 |  | 0.0159 |  | 46.9 |  | < .001 |  | 0.848 |  | |
| Factor 4 |  | ASKU1 |  | 0.721 |  | 0.0144 |  | 50.2 |  | < .001 |  | 0.845 | 76% | |
|  |  | ASKU2 |  | 0.758 |  | 0.0138 |  | 55.1 |  | < .001 |  | 0.895 |  | |
|  |  | ASKU3 |  | 0.770 |  | 0.0146 |  | 52.6 |  | < .001 |  | 0.870 |  | |
