# Supplementary material for: New norm values of the brief resilience scale (BRS) from the German general population with new post-COVID-19 data
Source: BMC Psychol. 2024 Sep 27;12:499. doi: 10.1186/s40359-024-01995-0 (PMC11437619; doi:10.1186/s40359-024-01995-0)
Supplement: Supplementary file 2 — Supplementary Material 2 [file 40359_2024_1995_MOESM2_ESM.docx]

**Population-based norms of the BRS**

Table S5.

| *Percent rank and stanine values for the BRS in the total sample* | | |
| --- | --- | --- |
| Total sample (*n* = 2,249) | | |
| Mean scores | PR | Stanine |
| 1.0 | 0.2 | 1 |
| 1.1 | 0.4^1^ | 1 |
| 1.2 | 0.4 | 1 |
| 1.3 | 0.8 | 1 |
| 1.4 | 1.1^1^ | 1 |
| 1.5 | 1.1 | 1 |
| 1.6 | 1.6^1^ | 1 |
| 1.7 | 1.6 | 1 |
| 1.8 | 2.3 | 1 |
| 1.9 | 3.6^1^ | 1 |
| 2.0 | 3.6 | 1 |
| 2.1 | 5.4^1^ | 2 |
| 2.2 | 5.4 | 2 |
| 2.3 | 7.5 | 2 |
| 2.4 | 10.0^1^ | 2 |
| 2.5 | 10.0 | 2 |
| 2.6 | 13.3^1^ | 3 |
| 2.7 | 13.3 | 3 |
| 2.8 | 17.8 | 3 |
| 2.9 | 24.2^1^ | 4 |
| 3.0 | 24.2 | 4 |
| 3.1 | 32.3^1^ | 4 |
| 3.2 | 32.3 | 4 |
| 3.3 | 40.3 | 5 |
| 3.4 | 48.1^1^ | 5 |
| 3.5 | 48.1 | 5 |
| 3.6 | 56.0^1^ | 5 |
| 3.7 | 56.0 | 5 |
| 3.8 | 62.8 | 6 |
| 3.9 | 71.3^1^ | 6 |
| 4.0 | 71.3 | 6 |
| 4.1 | 78.8^1^ | 7 |
| 4.2 | 78.8 | 7 |
| 4.3 | 82.7 | 7 |
| 4.4 | 86.7^1^ | 7 |
| 4.5 | 86.7 | 7 |
| 4.6 | 90.1^1^ | 8 |
| 4.7 | 90.1 | 8 |
| 4.8 | 92.3 | 8 |
| 4.9 | 96.6^1^ | 9 |
| 5.0 | 96.6 | 9 |

*Note.* PR = Percent rank; ^1^ In case of empirically not available mean scores, the next high percent rank applies (e.g., percent rank 0.4 for the mean 1.1 for the total sample)

Table S6

| *Percent ranks and stanine values for the BRS stratified by age* | | | | | | | | | | | | | | | | |
| --- | --- | --- | --- | --- | --- | --- | --- | --- | --- | --- | --- | --- | --- | --- | --- | --- |
| Total sample  (*N* = 2,499) | | | <25 years  (*n* = 205) | | 25-34 years  (*n* = 403) | | 35-44 years  (*n* = 449) | | 45-54 years  (*n* = 412) | | 55-64 years  (*n* = 483) | | 65-74 years  (*n* = 341) | | > 74 years  (*n* = 206) | |
| Mean scores | PR | Stanine | PR | Stanine | PR | Stanine | PR | Stanine | PR | Stanine | PR | Stanine | PR | Stanine | PR | Stanine |
| 1.0 | 0.2 | 1 | 0.5^1^ | 1 | 0.4^1^ | 1 | 0.2^1^ | 1 | 0.2 | 1 | 0.6 | 1 | 0.3 | 1 | 0.7^1^ | 1 |
| 1.1 | 0.4^1^ | 1 | 0.5^1^ | 1 | 0.4^1^ | 1 | 0.2^1^ | 1 | 0.5^1^ | 1 | 1.5^1^ | 1 | 0.6^1^ | 1 | 0.7^1^ | 1 |
| 1.2 | 0.4 | 1 | 0.5^1^ | 1 | 0.4 | 1 | 0.2^1^ | 1 | 0.5 | 1 | 1.5 | 1 | 0.6 | 1 | 0.7^1^ | 1 |
| 1.3 | 0.8 | 1 | 0.5 | 1 | 0.7^1^ | 1 | 0.2^1^ | 1 | 0.7 | 1 | 2.1 | 1 | 1.0 | 1 | 0.7 | 1 |
| 1.4 | 1.1^1^ | 1 | 1.0^1^ | 1 | 0.7^1^ | 1 | 0.2^1^ | 1 | 1.6^1^ | 1 | 2.7^1^ | 1 | 1.6^1^ | 1 | 1.7^1^ | 1 |
| 1.5 | 1.1 | 1 | 1.0^1^ | 1 | 0.7 | 1 | 0.2 | 1 | 1.6^1^ | 1 | 2.7 | 1 | 1.6 | 1 | 1.7^1^ | 1 |
| 1.6 | 1.6^1^ | 1 | 1.0^1^ | 1 | 1.4^1^ | 1 | 0.4^1^ | 1 | 1.6^1^ | 1 | 3.3^1^ | 1 | 2.2^1^ | 1 | 1.7^1^ | 1 |
| 1.7 | 1.6 | 1 | 1.0 | 1 | 1.4^1^ | 1 | 0.4 | 1 | 1.6 | 1 | 3.3 | 1 | 2.2 | 1 | 1.7 | 1 |
| 1.8 | 2.3 | 1 | 2.4 | 1 | 1.4 | 1 | 0.7 | 1 | 2.8 | 1 | 4.0 | 2 | 2.9 | 1 | 3.4 | 1 |
| 1.9 | 3.6^1^ | 1 | 5.1^1^ | 2 | 1.9^1^ | 1 | 1.9^1^ | 1 | 4.1^1^ | 2 | 5.2^1^ | 2 | 4.1^1^ | 2 | 5.6^1^ | 2 |
| 2.0 | 3.6 | 1 | 5.1 | 2 | 1.9 | 1 | 1.9 | 1 | 4.1 | 2 | 5.2 | 2 | 4.1 | 2 | 5.6 | 2 |
| 2.1 | 5.4^1^ | 2 | 7.8^1^ | 2 | 2.8^1^ | 1 | 4.2^1^ | 2 | 5.1^1^ | 2 | 6.7^1^ | 2 | 6.0^1^ | 2 | 8.3^1^ | 2 |
| 2.2 | 5.4 | 2 | 7.8 | 2 | 2.8 | 1 | 4.2 | 2 | 5.1 | 2 | 6.7 | 2 | 6.0 | 2 | 8.3 | 2 |
| 2.3 | 7.5 | 2 | 10.7 | 2 | 5.4 | 2 | 5.8 | 2 | 6.3 | 2 | 8.9 | 2 | 8.2 | 2 | 11.4 | 3 |
| 2.4 | 10.0^1^ | 2 | 14.8^1^ | 3 | 8.5^1^ | 2 | 6.6^1^ | 2 | 8.3^1^ | 2 | 11.5^1^ | 3 | 11.0^1^ | 3 | 14.8^1^ | 3 |
| 2.5 | 10.0 | 2 | 14.8 | 3 | 8.5 | 2 | 6.6 | 2 | 8.3 | 2 | 11.5 | 3 | 11.0 | 3 | 14.8 | 3 |
| 2.6 | 13.3^1^ | 3 | 19.9^1^ | 3 | 11.7^1^ | 3 | 9.1^1^ | 2 | 11.3^1^ | 3 | 14.9^1^ | 3 | 14.8^1^ | 3 | 18.7^1^ | 3 |
| 2.7 | 13.3 | 3 | 19.9 | 3 | 11.7 | 3 | 9.1 | 2 | 11.3 | 3 | 14.9 | 3 | 14.8 | 3 | 18.7 | 3 |
| 2.8 | 17.8 | 3 | 23.8 | 4 | 15.4 | 3 | 13.1 | 3 | 16.8 | 3 | 19.2 | 3 | 20.2 | 3 | 22.6 | 4 |
| 2.9 | 24.2^1^ | 4 | 29.1^1^ | 4 | 20.5^1^ | 3 | 20.1^1^ | 3 | 24.4^1^ | 4 | 25.1^1^ | 4 | 27.9^1^ | 4 | 28.6^1^ | 4 |
| 3.0 | 24.2 | 4 | 29.1 | 4 | 20.5 | 3 | 20.1 | 3 | 24.4 | 4 | 25.1 | 4 | 27.9 | 4 | 28.6 | 4 |
| 3.1 | 32.3^1^ | 4 | 36.2^1^ | 4 | 27.8^1^ | 4 | 28.6^1^ | 4 | 34.2^1^ | 4 | 32.2^1^ | 4 | 36.5^1^ | 4 | 36.9^1^ | 4 |
| 3.2 | 32.3 | 4 | 36.2 | 4 | 27.8 | 4 | 28.6 | 4 | 34.2 | 4 | 32.2 | 4 | 36.5 | 4 | 36.9 | 4 |
| 3.3 | 40.3 | 5 | 43.9 | 5 | 34.9 | 4 | 34.7 | 4 | 43.3 | 5 | 39.4 | 4 | 46.5 | 5 | 45.1 | 5 |
| 3.4 | 48.1^1^ | 5 | 51.5^1^ | 5 | 43.1^1^ | 5 | 41.9^1^ | 5 | 50.9^1^ | 5 | 47.5^1^ | 5 | 55.6^1^ | 5 | 53.6^1^ | 5 |
| 3.5 | 48.1 | 5 | 51.5 | 5 | 43.1 | 5 | 41.9 | 5 | 50.9 | 5 | 47.5 | 5 | 55.6 | 5 | 53.6 | 5 |

*Note.* PR = Percent rank; ^1^ In case of empirically not available mean scores, the next high percent rank applies (e.g., percent rank 0.4 for the mean 1.1 for the total sample)

Table S6 *(continued)*

| *Percent ranks and stanine values for the BRS stratified by age* | | | | | | | | | | | | | | | | |
| --- | --- | --- | --- | --- | --- | --- | --- | --- | --- | --- | --- | --- | --- | --- | --- | --- |
| Total sample  (*N* = 2,499) | | | <25 years  (*n* = 205) | | 25-34 years  (*n* = 403) | | 35-44 years  (*n* = 449) | | 45-54 years  (*n* = 412) | | 55-64 years  (*n* = 483) | | 65-74 years  (*n* = 341) | | > 75 years  (*n* = 206) | |
| Mean scores | PR | Stanine | PR | Stanine | PR | Stanine | PR | Stanine | PR | Stanine | PR | Stanine | PR | Stanine | PR | Stanine |
| 3.6 | 56.0^1^ | 5 | 57.3^1^ | 5 | 52.0^1^ | 5 | 51.0^1^ | 5 | 57.2^1^ | 5 | 55.6^1^ | 5 | 62.9^1^ | 6 | 62.1^1^ | 6 |
| 3.7 | 56.0 | 5 | 57.3 | 5 | 52.0 | 5 | 51.0 | 5 | 57.2 | 5 | 55.6 | 5 | 62.9 | 6 | 62.1 | 6 |
| 3.8 | 62.8 | 6 | 63.4 | 6 | 59.1 | 5 | 59.1^1^ | 5 | 63.6 | 6 | 62.2 | 6 | 69.2 | 6 | 67.7 | 6 |
| 3.9 | 71.3^1^ | 6 | 69.9^1^ | 6 | 67.8^1^ | 6 | 59.1 | 5 | 72.7^1^ | 6 | 70.4^1^ | 6 | 76.7^1^ | 6 | 76.7^1^ | 6 |
| 4.0 | 71.3 | 6 | 69.9 | 6 | 67.8 | 6 | 68.7^1^ | 6 | 72.7 | 6 | 70.4 | 6 | 76.7 | 6 | 76.7 | 6 |
| 4.1 | 78.8^1^ | 7 | 75.2^1^ | 6 | 74.5^1^ | 6 | 68.7 | 6 | 80.0^1^ | 7 | 78.1^1^ | 7 | 83.0^1^ | 7 | 86.4^1^ | 7 |
| 4.2 | 78.8 | 7 | 75.2 | 6 | 74.5 | 6 | 77.9 | 7 | 80.0 | 7 | 78.1 | 7 | 83.0 | 7 | 86.4 | 7 |
| 4.3 | 82.7 | 7 | 78.2^1^ | 7 | 78.3 | 7 | 83.1 | 7 | 83.1 | 7 | 82.3 | 7 | 86.5 | 7 | 90.3 | 8 |
| 4.4 | 86.7^1^ | 7 | 78.2 | 7 | 82.4^1^ | 7 | 87.4^1^ | 7 | 87.5^1^ | 7 | 86.3^1^ | 7 | 90.2^1^ | 8 | 93.0^1^ | 8 |
| 4.5 | 86.7 | 7 | 82.0 | 7 | 82.4 | 7 | 87.4 | 7 | 87.5 | 7 | 86.3 | 7 | 90.2 | 8 | 93.0 | 8 |
| 4.6 | 90.1^1^ | 8 | 85.7^1^ | 7 | 86.3^1^ | 7 | 91.0^1^ | 8 | 91.1^1^ | 8 | 89.4^1^ | 8 | 93.2^1^ | 8 | 95.2^1^ | 8 |
| 4.7 | 90.1 | 8 | 85.7 | 7 | 86.3 | 7 | 91.0 | 8 | 91.1 | 8 | 89.4 | 8 | 93.2 | 8 | 95.2 | 8 |
| 4.8 | 92.3 | 8 | 88.4 | 7 | 89.4 | 8 | 93.2 | 8 | 92.7 | 8 | 91.8 | 8 | 95.3 | 8 | 96.9 | 9 |
| 4.9 | 96.6^1^ | 9 | 95.2^1^ | 8 | 95.2^1^ | 8 | 97.1^1^ | 9 | 96.9^1^ | 9 | 96.6^1^ | 9 | 98.0^1^ | 9 | 98.8^1^ | 9 |
| 5.0 | 96.6 | 9 | 95.2 | 8 | 95.2 | 8 | 97.1 | 9 | 96.9 | 9 | 96.6 | 9 | 98.0 | 9 | 98.8 | 9 |

*Note.* PR = Percent rank; ^1^ In case of empirically not available mean scores, the next high percent rank applies (e.g., percent rank 0.4 for the mean 1.1 for the total sample)

Table S7

| *Percent ranks and stanine values for the BRS stratified by gender* | | | | | | | | | | |
| --- | --- | --- | --- | --- | --- | --- | --- | --- | --- | --- |
| Total sample  (*N* = 2,499) | | | | |  | Men  (*n* = 1,245) | |  | Women  (*n* = 1,254) | |
| Mean scores | | PR | Stanine | |  | PR | Stanine |  | PR | Stanine |
| 1.0 | 0.2 | | | 1 | | 0.2 | 1 | | 0.2 | 1 |
| 1.1 | 0.4^1^ | | | 1 | | 0.7^1^ | 1 | | 0.4^1^ | 1 |
| 1.2 | 0.4 | | | 1 | | 0.7 | 1 | | 0.4 | 1 |
| 1.3 | 0.8 | | | 1 | | 1.0 | 1 | | 0.8 | 1 |
| 1.4 | 1.1^1^ | | | 1 | | 1.1^1^ | 1 | | 1.1^1^ | 1 |
| 1.5 | 1.1 | | | 1 | | 1.1 | 1 | | 1.1 | 1 |
| 1.6 | 1.6^1^ | | | 1 | | 1.3^1^ | 1 | | 1.6^1^ | 1 |
| 1.7 | 1.6 | | | 1 | | 1.3 | 1 | | 1.6 | 1 |
| 1.8 | 2.3 | | | 1 | | 1.7 | 1 | | 2.3 | 1 |
| 1.9 | 3.6^1^ | | | 1 | | 2.4^1^ | 1 | | 3.6^1^ | 1 |
| 2.0 | 3.6 | | | 1 | | 2.4 | 1 | | 3.6 | 1 |
| 2.1 | 5.4^1^ | | | 2 | | 3.3^1^ | 1 | | 5.4^1^ | 2 |
| 2.2 | 5.4 | | | 2 | | 3.3 | 1 | | 5.4 | 2 |
| 2.3 | 7.5 | | | 2 | | 4.3 | 2 | | 7.5 | 2 |
| 2.4 | 10.0^1^ | | | 2 | | 5.8^1^ | 2 | | 10.0^1^ | 2 |
| 2.5 | 10.0 | | | 2 | | 5.8 | 2 | | 10.0 | 2 |
| 2.6 | 13.3^1^ | | | 3 | | 8.5^1^ | 2 | | 13.3^1^ | 3 |
| 2.7 | 13.3 | | | 3 | | 8.5 | 2 | | 13.3 | 3 |
| 2.8 | 17.8 | | | 3 | | 12.5 | 3 | | 17.8 | 3 |
| 2.9 | 24.2^1^ | | | 4 | | 18.5^1^ | 3 | | 24.2^1^ | 4 |
| 3.0 | 24.2 | | | 4 | | 18.5 | 3 | | 24.2 | 4 |
| 3.1 | 32.3^1^ | | | 4 | | 25.9^1^ | 4 | | 32.3^1^ | 4 |
| 3.2 | 32.3 | | | 4 | | 25.9 | 4 | | 32.3 | 4 |
| 3.3 | 40.3 | | | 5 | | 32.7 | 4 | | 40.2 | 5 |
| 3.4 | 48.1^1^ | | | 5 | | 40.4^1^ | 5 | | 48.1^1^ | 5 |
| 3.5 | 48.1 | | | 5 | | 40.4 | 5 | | 48.1 | 5 |
| 3.6 | 56.0^1^ | | | 5 | | 48.9^1^ | 5 | | 56.0^1^ | 5 |
| 3.7 | 56.0 | | | 5 | | 48.9 | 5 | | 56.0 | 5 |
| 3.8 | 62.8 | | | 6 | | 56.2 | 5 | | 62.8 | 6 |
| 3.9 | 71.3^1^ | | | 6 | | 66.0^1^ | 6 | | 71.3^1^ | 6 |
| 4.0 | 71.3 | | | 6 | | 66.0 | 6 | | 71.3 | 6 |
| 4.1 | 78.8^1^ | | | 7 | | 74.9^1^ | 6 | | 78.8^1^ | 7 |
| 4.2 | 78.8 | | | 7 | | 74.9 | 6 | | 78.8 | 7 |
| 4.3 | 82.7 | | | 7 | | 79.3 | 7 | | 82.7 | 7 |
| 4.4 | 86.7^1^ | | | 7 | | 83.8^1^ | 7 | | 86.7^1^ | 7 |
| 4.5 | 86.7 | | | 7 | | 83.8 | 7 | | 86.7 | 7 |
| 4.6 | 90.1^1^ | | | 8 | | 87.7^1^ | 7 | | 90.1 | 8 |
| 4.7 | 90.1 | | | 8 | | 87.7 | 7 | | 92.3^1^ | 8 |
| 4.8 | 92.3 | | | 8 | | 90.2 | 8 | | 92.3 | 8 |
| 4.9 | 96.6^1^ | | | 9 | | 95.7^1^ | 8 | | 96.6^1^ | 9 |
| 5.0 | 96.6 | | | 9 | | 95.7 | 8 | | 96.6 | 9 |

*Note.* PR = Percent rank; ^1^ In case of empirically not available mean scores, the next high percent rank applies (e.g., percent rank 0.4 for the mean 1.1 for the total sample).

Table S8

| *Percent ranks and stanine values for the BRS stratified by age and gender – Men* | | | | | | | | | | | | | | | | | |
| --- | --- | --- | --- | --- | --- | --- | --- | --- | --- | --- | --- | --- | --- | --- | --- | --- | --- |
| Men – all age groups  (*n* = 1245) | | | | <25 years  (*n* = 95) | | 25-34 years  (*n* = 203) | | 35-44 years  (*n* = 229) | | 45-54 years  (*n* = 204) | | 55-64 years  (*n* = 244) | | 65-74 years  (*n* = 174) | | > 75 years  (*n* = 96) | |
| Mean scores | PR | Stanine | | PR | Stanine | PR | Stanine | PR | Stanine | PR | Stanine | PR | Stanine | PR | Stanine | PR | Stanine |
| 1.0 | 0.2 | | 1 | 1.1^1^ | 1 | 0.5^1^ | 1 | 0.4^1^ | 1 | 0.5^1^ | 1 | 1.0 | 1 | 0.6 | 1 | 1.6^1^ | 1 |
| 1.1 | 0.7^1^ | | 1 | 1.1^1^ | 1 | 0.5^1^ | 1 | 0.4^1^ | 1 | 0.5^1^ | 1 | 2.5^1^ | 1 | 1.2^1^ | 1 | 1.6^1^ | 1 |
| 1.2 | 0.7 | | 1 | 1.1^1^ | 1 | 0.5 | 1 | 0.4^1^ | 1 | 0.5 | 1 | 2.5 | 1 | 1.2 | 1 | 1.6^1^ | 1 |
| 1.3 | 1.0 | | 1 | 1.1^1^ | 1 | 1.0^1^ | 1 | 0.4^1^ | 1 | 1.0^1^ | 1 | 3.5 | 1 | 1.7 | 1 | 1.6^1^ | 1 |
| 1.4 | 1.1^1^ | | 1 | 1.1^1^ | 1 | 1.0^1^ | 1 | 0.4^1^ | 1 | 1.0^1^ | 1 | 4.3^1^ | 2 | 1.7^1^ | 1 | 1.6^1^ | 1 |
| 1.5 | 1.1 | | 1 | 1.1^1^ | 1 | 1.0^1^ | 1 | 0.4 | 1 | 1.0^1^ | 1 | 4.3^1^ | 2 | 1.7 | 1 | 1.6^1^ | 1 |
| 1.6 | 1.3^1^ | | 1 | 1.1^1^ | 1 | 1.0^1^ | 1 | 0.9^1^ | 1 | 1.0^1^ | 1 | 4.3^1^ | 2 | 1.7^1^ | 1 | 1.6^1^ | 1 |
| 1.7 | 1.3 | | 1 | 1.1 | 1 | 1.0^1^ | 1 | 0.9^1^ | 1 | 1.0 | 1 | 4.3 | 2 | 1.7^1^ | 1 | 1.6^1^ | 1 |
| 1.8 | 1.7 | | 1 | 1.6^1^ | 1 | 1.0^1^ | 1 | 0.9 | 1 | 1.7^1^ | 1 | 4.9 | 2 | 1.7^1^ | 1 | 1.6 | 1 |
| 1.9 | 2.4^1^ | | 1 | 1.6 | 1 | 1.0^1^ | 1 | 1.7^1^ | 1 | 1.7^1^ | 1 | 5.3^1^ | 2 | 1.7^1^ | 1 | 3.1^1^ | 1 |
| 2.0 | 2.4 | | 1 | 4.2 | 2 | 1.0 | 1 | 1.7 | 1 | 1.7 | 1 | 5.3 | 2 | 1.7 | 1 | 3.1 | 1 |
| 2.1 | 3.3^1^ | | 1 | 6.3^1^ | 2 | 1.5^1^ | 1 | 3.3^1^ | 1 | 2.5^1^ | 1 | 5.7^1^ | 2 | 2.6^1^ | 1 | 4.7^1^ | 2 |
| 2.2 | 3.3 | | 1 | 6.3 | 2 | 1.5 | 1 | 3.3 | 1 | 2.5^1^ | 1 | 5.7 | 2 | 2.6 | 1 | 4.7 | 2 |
| 2.3 | 4.3 | | 2 | 9.5 | 2 | 3.5 | 1 | 4.4^1^ | 2 | 2.5 | 1 | 7.0^1^ | 2 | 3.7 | 1 | 6.8^1^ | 2 |
| 2.4 | 5.8^1^ | | 2 | 11.6^1^ | 3 | 6.9^1^ | 2 | 4.4^1^ | 2 | 3.2^1^ | 1 | 7.0^1^ | 2 | 5.7^1^ | 2 | 6.8^1^ | 2 |
| 2.5 | 5.8 | | 2 | 11.6 | 3 | 6.9 | 2 | 4.4 | 2 | 3.2 | 1 | 7.0 | 2 | 5.7 | 2 | 6.8 | 2 |
| 2.6 | 8.5^1^ | | 2 | 14.8^1^ | 3 | 10.1^1^ | 2 | 6.1^1^ | 2 | 5.4^1^ | 2 | 9.6^1^ | 2 | 8.9^1^ | 2 | 10.4^1^ | 2 |
| 2.7 | 8.5 | | 2 | 14.8 | 3 | 10.1 | 2 | 6.1 | 2 | 5.4 | 2 | 9.6 | 2 | 8.9 | 2 | 10.4 | 2 |
| 2.8 | 12.5 | | 3 | 18.4 | 3 | 12.8 | 3 | 9.4^1^ | 2 | 11.0 | 3 | 13.9 | 3 | 13.8 | 3 | 13.5 | 3 |
| 2.9 | 18.5^1^ | | 3 | 23.7^1^ | 4 | 16.3^1^ | 3 | 9.4 | 2 | 18.6^1^ | 3 | 20.1^1^ | 3 | 21.8^1^ | 3 | 16.7^1^ | 3 |
| 3.0 | 18.5 | | 3 | 23.7 | 4 | 16.3 | 3 | 16.4 | 3 | 18.6 | 3 | 20.1 | 3 | 21.8 | 3 | 16.7 | 3 |
| 3.1 | 25.9^1^ | | 4 | 30.5^1^ | 4 | 22.7^1^ | 3 | 24.0^1^ | 4 | 27.5^1^ | 4 | 26.2^1^ | 4 | 30.7^1^ | 4 | 22.9^1^ | 3 |
| 3.2 | 25.9 | | 4 | 30.5 | 4 | 22.7 | 3 | 24.0 | 4 | 27.5 | 4 | 26.2 | 4 | 30.7 | 4 | 22.9 | 3 |
| 3.3 | 32.7 | | 4 | 36.8 | 4 | 29.8 | 4 | 28.8 | 4 | 35.3^1^ | 4 | 32.8 | 4 | 39.4^1^ | 4 | 29.2^1^ | 4 |
| 3.4 | 40.4^1^ | | 5 | 43.2^1^ | 5 | 37.9^1^ | 4 | 36.3^1^ | 4 | 35.3 | 4 | 41.4^1^ | 5 | 39.4 | 4 | 37.0^1^ | 4 |
| 3.5 | 40.4 | | 5 | 43.2 | 5 | 37.9 | 4 | 36.3 | 4 | 41.9 | 5 | 41.4 | 5 | 47.0 | 5 | 37.0 | 4 |
| 3.6 | 48.9^1^ | | 5 | 49.0^1^ | 5 | 47.0^1^ | 5 | 46.3^1^ | 5 | 48.3^1^ | 5 | 49.8^1^ | 5 | 56.6^1^ | 5 | 47.4^1^ | 5 |

*Note.* PR = Percent rank; ^1^ In case of empirically not available mean scores, the next high percent rank applies (e.g., percent rank 0.4 for the mean 1.1 for the total sample)

Table S8 *(continued)*

| *Percent ranks and stanine values for the BRS stratified by age and gender – Men* | | | | | | | | | | | | | | | | | |
| --- | --- | --- | --- | --- | --- | --- | --- | --- | --- | --- | --- | --- | --- | --- | --- | --- | --- |
| Men – all age groups  (*n* = 1245) | | | | <25 years  (*n* = 95) | | 25-34 years  (*n* = 203) | | 35-44 years  (*n* = 229) | | 45-54 years  (*n* = 204) | | 55-64 years  (*n* = 244) | | 65-74 years  (*n* = 174) | | > 75 years  (*n* = 96) | |
| Mean scores | PR | Stanine | | PR | Stanine | PR | Stanine | PR | Stanine | PR | Stanine | PR | Stanine | PR | Stanine | PR | Stanine |
| 3.7 | 48.9 | | 5 | 49.0 | 5 | 47.0 | 5 | 46.3 | 5 | 48.3 | 5 | 48.9 | 5 | 56.6 | 5 | 47.4 | 5 |
| 3.8 | 56.2 | | 5 | 55.8 | 5 | 54.2 | 5 | 54.4 | 5 | 54.9 | 5 | 57.2 | 5 | 64.4 | 6 | 53.7^1^ | 5 |
| 3.9 | 66.0^1^ | | 6 | 63.2^1^ | 6 | 63.3^1^ | 6 | 64.4^1^ | 6 | 66.2^1^ | 6 | 66.6^1^ | 6 | 73.6^1^ | 6 | 66.2^1^ | 6 |
| 4.0 | 66.0 | | 6 | 63.2 | 6 | 63.3 | 6 | 64.4 | 6 | 66.2 | 6 | 66.6 | 6 | 73.6 | 6 | 66.2 | 6 |
| 4.1 | 74.9^1^ | | 6 | 69.0^1^ | 6 | 70.2^1^ | 6 | 74.9^1^ | 6 | 75.3^1^ | 6 | 74.8^1^ | 6 | 81.3^1^ | 7 | 81.8^1^ | 7 |
| 4.2 | 74.9 | | 6 | 69.0 | 6 | 70.2 | 6 | 74.9 | 6 | 75.3 | 6 | 74.8 | 6 | 81.3 | 7 | 81.8 | 7 |
| 4.3 | 79.3 | | 7 | 72.6 | 6 | 73.4 | 6 | 81.2^1^ | 7 | 78.7 | 7 | 79.3 | 7 | 85.1 | 7 | 87.5 | 7 |
| 4.4 | 83.8^1^ | | 7 | 76.8^1^ | 6 | 78.1^1^ | 7 | 81.2 | 7 | 84.3^1^ | 7 | 83.6^1^ | 7 | 88.5^1^ | 7 | 90.6^1^ | 8 |
| 4.5 | 83.8 | | 7 | 76.8 | 6 | 78.1 | 7 | 86.3 | 7 | 84.3 | 7 | 83.6 | 7 | 88.5 | 7 | 90.6 | 8 |
| 4.6 | 87.7^1^ | | 7 | 81.6^1^ | 7 | 83.3^1^ | 7 | 90.2^1^ | 8 | 89.5^1^ | 8 | 87.1^1^ | 7 | 91.1^1^ | 8 | 93.8^1^ | 8 |
| 4.7 | 87.7 | | 7 | 81.6^1^ | 7 | 83.3 | 7 | 90.2 | 8 | 89.5 | 8 | 87.1 | 7 | 91.1 | 8 | 93.8 | 8 |
| 4.8 | 90.2 | | 8 | 81.6 | 7 | 86.7 | 7 | 93.0 | 8 | 91.9 | 8 | 89.6 | 8 | 92.8 | 8 | 95.8 | 8 |
| 4.9 | 95.7^1^ | | 8 | 92.1^1^ | 8 | 93.8^1^ | 8 | 97.2^1^ | 9 | 96.8^1^ | 9 | 95.5^1^ | 8 | 96.8^1^ | 9 | 98.4^1^ | 9 |
| 5.0 | 95.7 | | 8 | 92.1 | 8 | 93.8 | 8 | 97.2 | 9 | 96.8 | 9 | 95.5 | 8 | 96.8 | 9 | 98.4 | 9 |

*Note.* PR = Percent rank; ^1^ In case of empirically not available mean scores, the next high percent rank applies (e.g., percent rank 0.7 for the mean 1.1 for the total sample)

Table S9

| *Percent ranks and stanine values for the BRS stratified by age and gender – Women* | | | | | | | | | | | | | | | | | |
| --- | --- | --- | --- | --- | --- | --- | --- | --- | --- | --- | --- | --- | --- | --- | --- | --- | --- |
| Women – all age groups  (*n* = 1254) | | | | <25 years  (*n* = 110) | | 25-34 years  (*n* = 200) | | 35-44 years  (*n* = 220) | | 45-54 years  (*n* = 208) | | 55-64 years  (*n* = 239) | | 65-74 years  (*n* = 167) | | > 74 years  (*n* = 110) | |
| Mean scores | PR | | Stanine | PR | Stanine | PR | Stanine | PR | Stanine | PR | Stanine | PR | Stanine | PR | Stanine | PR | Stanine |
| 1.0 | 0.2 | 1 | | 0.9^1^ | 1 | 0.5^1^ | 1 | 0.5^1^ | 1 | 0.5 | 1 | 0.4^1^ | 1 | 0.9^1^ | 1 | 1.4^1^ | 1 |
| 1.1 | 0.4^1^ | 1 | | 0.9^1^ | 1 | 0.5^1^ | 1 | 0.5^1^ | 1 | 1.0^1^ | 1 | 0.8^1^ | 1 | 0.9^1^ | 1 | 1.4^1^ | 1 |
| 1.2 | 0.4 | 1 | | 0.9^1^ | 1 | 0.5 | 1 | 0.5^1^ | 1 | 1.0^1^ | 1 | 0.8^1^ | 1 | 0.9^1^ | 1 | 1.4^1^ | 1 |
| 1.3 | 0.8 | 1 | | 0.9 | 1 | 1.3^1^ | 1 | 0.5^1^ | 1 | 1.0 | 1 | 0.8 | 1 | 0.9 | 1 | 1.4 | 1 |
| 1.4 | 1.1^1^ | 1 | | 2.7^1^ | 1 | 1.3^1^ | 1 | 0.5^1^ | 1 | 2.4^1^ | 1 | 1.7^1^ | 1 | 2.1^1^ | 1 | 3.2^1^ | 1 |
| 1.5 | 1.1 | 1 | | 2.7^1^ | 1 | 1.3 | 1 | 0.5^1^ | 1 | 2.4^1^ | 1 | 1.7 | 1 | 2.1 | 1 | 3.2^1^ | 1 |
| 1.6 | 1.6^1^ | 1 | | 2.7^1^ | 1 | 2.3^1^ | 1 | 0.5^1^ | 1 | 2.4^1^ | 1 | 2.5^1^ | 1 | 3.3^1^ | 1 | 3.2^1^ | 1 |
| 1.7 | 1.6 | 1 | | 2.7^1^ | 1 | 2.3^1^ | 1 | 0.5 | 1 | 2.4 | 1 | 2.5 | 1 | 3.3 | 1 | 3.2 | 1 |
| 1.8 | 2.3 | 1 | | 2.7 | 1 | 2.3 | 1 | 2.3^1^ | 1 | 4.6 | 2 | 3.4 | 1 | 4.8 | 2 | 5.5 | 2 |
| 1.9 | 3.6^1^ | 1 | | 6.4^1^ | 2 | 3.0^1^ | 1 | 2.3^1^ | 1 | 6.7^1^ | 2 | 5.2^1^ | 2 | 6.9^1^ | 2 | 8.2^1^ | 2 |
| 2.0 | 3.6 | 1 | | 6.4 | 2 | 3.0 | 1 | 2.3 | 1 | 6.7 | 2 | 5.2 | 2 | 6.9 | 2 | 8.2 | 2 |
| 2.1 | 5.4^1^ | 2 | | 9.6^1^ | 2 | 4.5^1^ | 2 | 5.5^1^ | 2 | 8.2^1^ | 2 | 7.8^1^ | 2 | 9.9^1^ | 2 | 11.8^1^ | 3 |
| 2.2 | 5.4 | 2 | | 9.6 | 2 | 4.5 | 2 | 5.5 | 2 | 8.2 | 2 | 7.8 | 2 | 9.9 | 2 | 11.8 | 3 |
| 2.3 | 7.5 | 2 | | 12.3 | 3 | 7.8 | 2 | 7.7 | 2 | 10.3 | 2 | 12.1 | 3 | 13.2 | 3 | 16.8 | 3 |
| 2.4 | 10.0^1^ | 2 | | 18.2^1^ | 3 | 10.5^1^ | 2 | 9.1^1^ | 2 | 13.5^1^ | 3 | 16.3^1^ | 3 | 16.8^1^ | 3 | 22.3^1^ | 3 |
| 2.5 | 10.0 | 2 | | 18.2 | 3 | 10.5 | 2 | 9.1 | 2 | 13.5 | 3 | 16.3 | 3 | 16.8 | 3 | 22.3 | 3 |
| 2.6 | 13.3^1^ | 3 | | 25.0^1^ | 4 | 13.8^1^ | 3 | 12.5^1^ | 2 | 17.3^1^ | 3 | 20.5^1^ | 3 | 21.3^1^ | 3 | 26.4^1^ | 4 |
| 2.7 | 13.3 | 3 | | 25.0 | 4 | 13.8^1^ | 3 | 12.5 | 2 | 17.3 | 3 | 20.5 | 3 | 21.3 | 3 | 26.4 | 4 |
| 2.8 | 17.8 | 3 | | 29.1 | 4 | 18.5 | 3 | 17.3 | 3 | 22.6 | 3 | 24.7 | 4 | 27.3 | 4 | 30.9 | 4 |
| 2.9 | 24.2^1^ | 4 | | 34.6^1^ | 4 | 25.3^1^ | 4 | 24.3^1^ | 4 | 30.3^1^ | 4 | 30.3^1^ | 4 | 34.4^1^ | 4 | 39.5^1^ | 4 |
| 3.0 | 24.2 | 4 | | 34.6 | 4 | 25.3 | 4 | 24.3 | 4 | 30.3 | 4 | 30.3 | 4 | 34.4 | 4 | 39.5 | 4 |
| 3.1 | 32.3^1^ | 4 | | 41.9^1^ | 5 | 33.5^1^ | 4 | 33.6^1^ | 4 | 41.1^1^ | 5 | 38.5^1^ | 4 | 42.8^1^ | 5 | 49.6^1^ | 5 |
| 3.2 | 32.3 | 4 | | 41.9 | 5 | 33.5 | 4 | 33.6 | 4 | 41.1 | 5 | 38.3 | 4 | 42.8 | 5 | 49.6 | 5 |
| 3.3 | 40.2 | 5 | | 50.9 | 5 | 40.8 | 5 | 41.1 | 5 | 51.7 | 5 | 46.4 | 5 | 54.2 | 5 | 59.6^1^ | 5 |
| 3.4 | 48.1^1^ | 5 | | 59.6^1^ | 5 | 49.0^1^ | 5 | 48.3^1^ | 5 | 59.9^1^ | 5 | 54.0^1^ | 5 | 63.8^1^ | 6 | 59.6 | 5 |
| 3.5 | 48.1 | 5 | | 59.6 | 5 | 49.0 | 5 | 48.2 | 5 | 59.9 | 5 | 54.0 | 5 | 63.8 | 6 | 68.6 | 6 |

*Note.* PR = Percent rank; ^1^ In case of empirically not available mean scores, the next high percent rank applies (e.g., percent rank 0.4 for the mean 1.1 for the total sample)

Table S9 *(continued)*

| *Percent ranks and stanine values for the BRS stratified by age and gender – Women* | | | | | | | | | | | | | | | | |
| --- | --- | --- | --- | --- | --- | --- | --- | --- | --- | --- | --- | --- | --- | --- | --- | --- |
| Women – all age groups  (*n* = 1254) | | | <25 years  (*n* = 110) | | 25-34 years  (*n* = 200) | | 35-44 years  (*n* = 220) | | 45-54 years  (*n* = 208) | | 55-64 years  (*n* = 239) | | 65-74 years  (*n* = 167) | | > 74 years  (*n* = 110) | |
| Mean scores | PR | Stanine | PR | Stanine | PR | Stanine | PR | Stanine | PR | Stanine | PR | Stanine | PR | Stanine | PR | Stanine |
| 3.6 | 56.0^1^ | 5 | 65.5^1^ | 6 | 57.5^1^ | 5 | 56.1^1^ | 5 | 66.4^1^ | 6 | 61.7^1^ | 6 | 69.8^1^ | 6 | 75.5^1^ | 6 |
| 3.7 | 56.0 | 5 | 65.5 | 6 | 57.5 | 5 | 56.1 | 5 | 66.4 | 6 | 61.7 | 6 | 69.8 | 6 | 75.5 | 6 |
| 3.8 | 62.8 | 6 | 70.9 | 6 | 64.5 | 6 | 64.1 | 6 | 72.4 | 6 | 67.6 | 6 | 74.6 | 6 | 80.5 | 7 |
| 3.9 | 71.3^1^ | 6 | 76.8^1^ | 6 | 72.8^1^ | 6 | 73.2^1^ | 6 | 79.3^1^ | 7 | 74.5^1^ | 6 | 80.2^1^ | 7 | 86.4^1^ | 7 |
| 4.0 | 71.3 | 6 | 76.8 | 6 | 72.8 | 6 | 73.2 | 6 | 79.3 | 7 | 74.5 | 6 | 80.2 | 7 | 86.4 | 7 |
| 4.1 | 78.8^1^ | 7 | 81.8^1^ | 7 | 79.5^1^ | 7 | 81.1^1^ | 7 | 84.9^1^ | 7 | 81.6^1^ | 7 | 85.0^1^ | 7 | 90.0^1^ | 8 |
| 4.2 | 78.8 | 7 | 81.8 | 7 | 79.5 | 7 | 81.1 | 7 | 84.9 | 7 | 81.6 | 7 | 85.0 | 7 | 90.0 | 8 |
| 4.3 | 82.7 | 7 | 84.1 | 7 | 83.8 | 7 | 85.2 | 7 | 87.5^1^ | 7 | 85.6 | 7 | 88.3 | 7 | 93.2 | 8 |
| 4.4 | 86.7^1^ | 7 | 87.7^1^ | 7 | 87.0^1^ | 7 | 88.9^1^ | 7 | 87.5 | 7 | 89.3^1^ | 8 | 92.2^1^ | 8 | 95.5^1^ | 8 |
| 4.5 | 86.7 | 7 | 87.7 | 7 | 87.0 | 7 | 88.9 | 7 | 90.9 | 8 | 89.3 | 8 | 92.2 | 8 | 95.5 | 8 |
| 4.6 | 90.1 | 8 | 92.3^1^ | 8 | 89.5^1^ | 8 | 92.1^1^ | 8 | 93.0^1^ | 8 | 92.1^1^ | 8 | 95.8^1^ | 8 | 96.8^1^ | 9 |
| 4.7 | 92.3^1^ | 8 | 92.3 | 8 | 89.5 | 8 | 92.1 | 8 | 93.0 | 8 | 92.1 | 8 | 95.8 | 8 | 96.8 | 9 |
| 4.8 | 92.3 | 8 | 95.5 | 8 | 92.3 | 8 | 93.6 | 8 | 93.8 | 8 | 94.4 | 8 | 98.2 | 9 | 98.2 | 9 |
| 4.9 | 96.6^1^ | 9 | 98.6^1^ | 9 | 96.8^1^ | 9 | 97.3^1^ | 9 | 97.1^1^ | 9 | 97.9^1^ | 9 | 99.4^1^ | 9 | 99.6^1^ | 9 |
| 5.0 | 96.6 | 9 | 98.6 | 9 | 96.8 | 9 | 97.3 | 9 | 97.1 | 9 | 97.9 | 9 | 99.4 | 9 | 99.6 | 9 |

*Note.* PR = Percent rank; ^1^ In case of empirically not available mean scores, the next high percent rank applies (e.g., percent rank 0.4 for the mean 1.1 for the total sample)
